# Parkinson’s Disease Subtypes Show a Specific Link between Dopaminergic and Glucose Metabolism in the Striatum

**DOI:** 10.1371/journal.pone.0096629

**Published:** 2014-05-21

**Authors:** Carsten Eggers, Frank Schwartz, David J. Pedrosa, Lutz Kracht, Lars Timmermann

**Affiliations:** 1 Department of Neurology, University Hospital of Cologne, Cologne, Germany; 2 Max-Planck-Institute for Neurological Research, Cologne, Germany; University of Ulm, Germany

## Abstract

**Background:**

Previous studies have shown different clinical and imaging pattern in tremordominant and akinetic-rigid Parkinson’s disease (PD) subtypes. The association between dopaminergic and glucose metabolism has in contrast not been investigated yet. Therefore, this study compared PD subtypes with respect to clinical and imaging findings with the aim of establishing a relationship between clinical subtypes, dopamine and glucose metabolism.

**Methods:**

Two groups of a total of 64 idiopathic PD patients (42 male, 22 female, mean age 56±10.9 years) were analysed: akinetic-rigid (AR, n = 32) and tremor-dominant (TD, n = 32) patients. Both were compared with respect to differential involvement of local striatal dopamine and glucose metabolism using [^18^F]-fluoro-L-dopa (F-dopa) and [^18^F]-fluorodeoxyglucose (FDG)-PET.

**Results:**

The analysis of PD subgroups showed significant differences in the F-dopa uptake in the anterior putamen. Using the results of the local striatal dopamine difference as a volume of interest for the FDG-analysis, analysis of AR patients revealed a significantly lower normalised cerebral metabolic rate of glucose (nCMRGlc) within the ventral striatum.

**Conclusions:**

The dual tracer study illlustrates clear differences between TD and AR subtypes in the ventral striatum. In accordance with previous FP-CIT-SPECT studies, it discloses congruent results for the presynaptic dopaminergic system and extends the knowledge about an additional involvement of local metabolic activity in the caudate and anterior putamen. The findings corroborate the specific role of distinct PD subtypes within the cerebello-thalamo-cortical-circuits. Multitracer PET imaging may thus enhance the knowledge about the clinical segregation into subtypes.

## Introduction

Parkinson’s disease (PD) patients differ concerning the clinical progress, symptom severity and symptom onset [1]–[3]. The well-known subtyping into akinetic-rigid, tremordominant and equivalent subtypes was recently amended by four large clusters of symptoms, e.g. patients with the main cluster profiles “old age-at-onset and rapid disease progression” and “young age-at-onset and slow disease progression” [4]. These results of PD subtyping have, however, not been transferred into clinical routine and large prospective cohorts confirming these data sets are pending. Besides, Rosenberg-Katz and coworkers demonstrated a gray matter atrophy in motor-related regions in postural-instability gait difficulty (PIGD) subtypes [5], which can be seen as a counterpart of akinetic-rigid patients, whereas white matter hyperintensities could not be assigned to one PD subtype [6]. Hence, current literature refers mainly to the standard classification.

Previously, our group demonstrated distinct differences in the dopaminergic system in classical PD subtypes both at baseline [7] and in the course of the disease [8]. We could prove a considerable clinical and in-vivo progression in terms of dopaminergic decline of akinetic-rigid patients over time, whereas tremordominant patients had a relatively stable course.

In this context and regarding the glucose metabolism, the group of Eidelberg developed a distinct pattern for tremor and rigidity in PD, the Parkinson’s Diseases Related Pattern (PDRP) [9] and Parkinson’s Disease Tremor Pattern (PDTP) [10], respectively. Both patterns showed an increased activity in caudate and putamen compared to healthy controls. However, when contrasting these two entities of PD, a partial overlap of these patterns arose for putaminal subregions. Anyway, the overall striatal glucose metabolism ( = caudate+putamen) for the AR/TD subtypes did not discriminate between the two patterns [10]. The importance of the striatum was also corroborated by Lozza et al. as they found inverse correlations for AR and TD between symptom severity and cerebral metabolic rate of glucose (cMRGlc) for putaminal regions [11].

Nevertheless, to our knowledge, little is known about the relationship between local dopaminergic uptake and the local neuronal function represented through the glucose metabolism in identical regions for specific PD subtypes. In order to refine the knowledge and the association between dopaminergic and glucose metabolism and about specific PD forms, we used [^18^F]-fluorodeoxyglucose (FDG) and [^18^F]-fluoro-L-dopa (F-dopa) PET scans in identical patients with specific subtypes of PD. We hypothesized subtype-specific binding patterns with reduced F-dopa and FDG uptake particularly in akinetic-rigid patients. The multimodal aspect of this study considered the local uptake of F-dopa and FDG in the same striatal regions identified as subtype-specific key structures.

## Methods

This retrospective analysis included 64 idiopathic PD patients (42 male, 22 female, mean age 56±10.9 years) who were divided into the subtypes akinetic-rigid (AR) and tremor-dominant (TD). Both groups were compared for striatal glucose and dopaminergic uptake. Due to the retrospective character of this study an ethical vote was not mandatory according to German federal laws. However, the ethics committee of the Medical Faculty of the University of Cologne approved the approach on request. Patients were not asked for informed consent as patient records/information was anonymized and de-identified prior to analysis.

### Patients

We evaluated a database of initially 227 patients who had two PET-scans using the tracers F-dopa and FDG including all patients with the diagnosis idiopathic PD and collected the following data out of patients’ charts: age, gender, disease duration, age at onset, predominantly affected body side, levodopa equivalent daily dose (LEDD, according to [12]), Hoehn and Yahr stage and subtype. As most patients’ charts did not include the UPDRS III, the assignment to distinct subtypes was based on the documented neurological examination as well as the classification in the patients’ charts. Usually, every patient was diagnosed with one subtype of the disease based on the clinical findings. As the Department of Neurology at the University Hospital of Cologne has a strong clinical focus and large experience in movement disorders we did not expect major weaknesses in these assignments. In case of doubt, patients were excluded for further analyses. Subjects with insufficient L-Dopa response, clinical signs of dementia, acute illnesses at the time of PET scanning, history of stroke or brain surgery were also excluded from further analysis. Diagnosis was reconfirmed two years after initial scans in order to exclude atypical parkinsonian syndromes or other diseases. With this proceeding, 64 AR and TD patients remained after thorough inspection of patients’ charts.

### Data Acquisition

The PET tracers included F-dopa to define the presynaptic F-dopa-uptake as measure for the nigrostriatal degeneration and FDG which represents the glucose metabolism. Scans were performed using a 24 detector ring scanner (ECAT EXACT HR, Siemens CTI, Knoxville, TN). PET scans were obtained according to previously published procedures [13]. Briefly, the procedures are described as following: During scanning procedures, subjects lay comfortably in a supine position in a room with dimmed lighting and low background noise. All antiparkinsonian medication was stopped at least 16 hours before starting PET registration. Data were acquired in a 3-D mode, subsequently reconstructed, including a correction for random coincidences, attenuation, and scatter. Both PET-scans (FDG & FDOPA) were registered on consecutive days with a minimum interval of 48 hours and a delay no longer than 13 days between scans.

[^18^F]-fluorodeoxyglucose (FDG): After the injection of 370 MBq of FDG, cerebral glucose metabolism was measured representing the regional metabolic activity. Arterialized venous blood sampling allowed absolute quantification for all subjects.

[^18^F]-fluoro-L-dopa (F-dopa): To avoid peripheral decarboxylation of F-dopa, 100 mg carbidopa was administered orally to the patients before the intravenous injection of 370 MBq F-dopa. Patients were scanned during 90 minutes, recording a dynamic series of nine 10-minute frames in a 3-dimensional mode.

Due to the applied techniques of using quantified FDG-PET data and semi-quantified, dynamic F-dopa PET-scans, this dataset achieves a high standard of data acquisition in order to reveal in-vivo processes.

### Data Analysis

**Figure 1:**
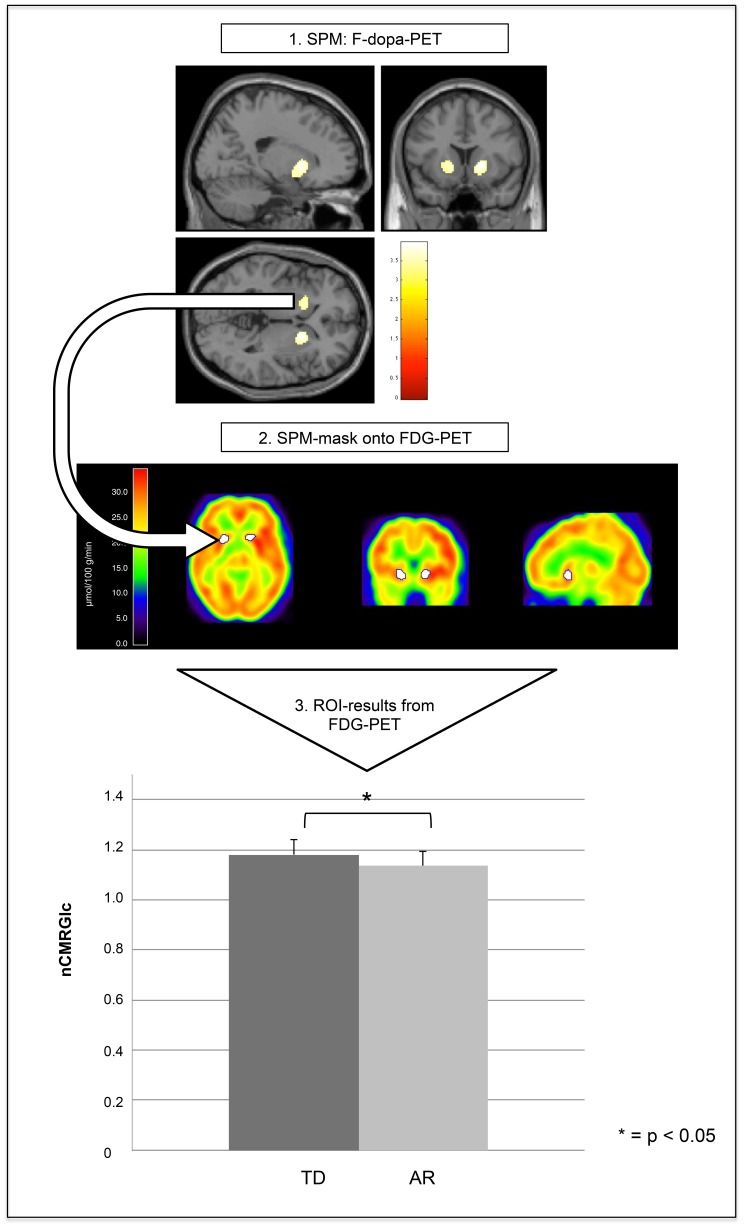
Flowchart of multimodal imaging. In a first step, AR and TD patients were contrasted for their respective F-dopa-uptake. Results show a more pronounced reduction in the ventral striatum for AR patients. Secondly, this SPM mask was used for evaluation of FDG-uptake in the corresponding region (white volumes of interest on the FDG-PETs). The boxplot demonstrates the significant differences in nCMRGlc between subtypes (* = p<0.05).

**Figure 2:**
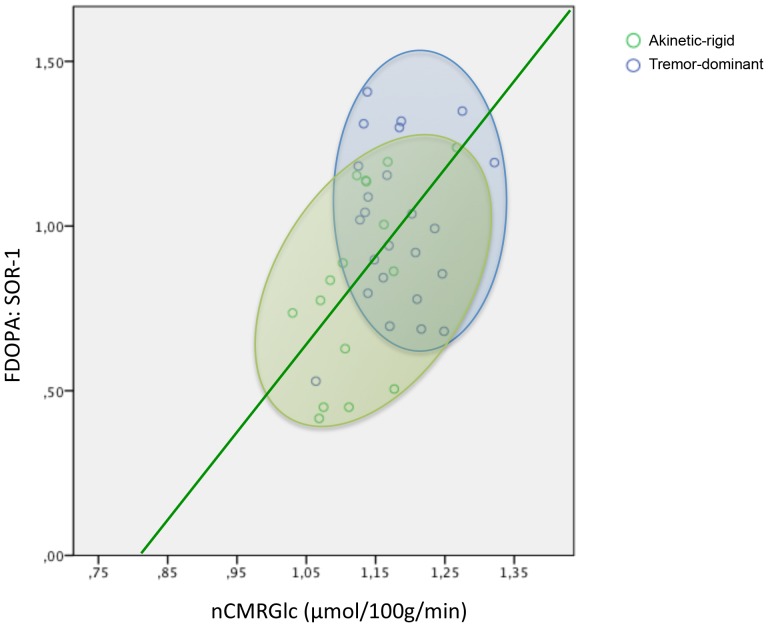
Regional glucose (nCMRGlc) and F-dopa (SOR-1) uptake in a striatal volume of interest identified as subtype-specific for PD patients. The green regression line demonstrates the correlation of nCMRGlc and F-dopa-uptake for akinetic-rigid patients (*r* = 0.537, *p* = 0.032). For methodological considerations see File S1.

To define local differences of dopaminergic uptake between subgroups, we used a voxel-based approach (SPM). The consecutive regional difference between these subtypes was used as a three dimensional volume of interest (VOI) to identify the local glucose metabolism in the respective individual FDG-PET scans. For an overview of the methodology see figure 1. Furthermore, we contrasted the local glucose metabolism within this VOI with the according local dopaminergic uptake within this volume (see figure 2).

#### Statistical parametric mapping

To compare the PET-scans with differing sizes and proportions in voxel-wise statistics, scans were preprocessed in SPM8 (The Wellcome Department of Cognitive Neurology, London, UK). Images were smoothed by a 10 mm full width at half maximum (FWHM) Gaussian filter and subsequently spatially normalized to a standard stereotactic space using an in-house created FP-CIT-template for the F-dopa scans (which has been used previously, see [8]) and the standard PET-template for FDG scans as provided in SPM.

According to our hypothesis, we expected a stronger decline of the dopaminergic uptake in the akinetic-rigid patients in striatal subregions. Thus, we used an a-priori hypothesis-driven “small volume approach” for the SPM analysis and compared statistically significant differences in a striatal mask encompassing the putamen and the caudate. This mask was generated with the wfu_pickatlas toolbox in SPM. We compared F-dopa PET scans of both subgroups using the t-test function in order to detect significant regional differences between TD and AR. False discovery rate was controlled with a cluster threshold of p<0.001. Results were considered significant for p-values lower than p<0.05 on the set-level. The corresponding regional difference was exported as a three-dimensional mask using the “save all clusters (binary)” function in SPM (see figure 1 step 1 & 2).

#### Global and regional metabolism

The global cerebral metabolic rates of glucose (gCMRGlc) was calculated as the averaged voxel activity in an elliptic region of interest encompassing all visible brain structures on a transaxial PET image plane transecting the striatum. The gCMRGlc between subgroups was compared using an independent samples t-test as distribution was parametric (SPSS 22.0 for Windows, IBM SPSS Statistics, IBM Corporation, Somers, NY). In order to reveal the regional CMRGlc (rCMRGlc) in the region for the dopaminergic difference between subtypes, the three-dimensional SPM mask was used on all individual normalized FDG-scans in an in-house created imaging tool (Vinci 4.2, www.nf.mpg.de/vinci3). This produced individual values for the rCMRGlc in this specific volume (see figure 1 step 2). Since we aimed at investigating local changes of energy metabolism independent from the variance of the global cerebral glucose metabolism, we subsequently normalized the rCMRGlc values by division with the individual global brain metabolism (gCMRGlc) in order to eliminate a systemic bias due to interindividual gCMRGlc differences (normalized CMRGlc = rCMRGlc/gCMRGlc).

The local dopaminergic uptake was determined using the dynamic PET scans between 80 and 90 min after injection. After spatial realignment and normalization, an occipital region of interest was placed on the last frame of the F-dopa scans. All scans (frame 80 to 90 min) of the patients were divided by the occipital uptake values and “1” was subtracted. This resulted in striatal-to-occiptial-ratios (SOR) -1 values for each subject. Due to a extremely low extrastriatal signal in K_i_-images of a graphical Patlak approach these images were not robustly normalized. Instead we used the SOR-1 technique which resulted in an ideal normalization process. For additional methodological considerations see [14]. To contrast the local, subtype-specific uptake for glucose and dopamine we used the same approach as for the FDG scans (export of SPM mask for the regional difference between subtypes, define the regional uptake for F-dopa scans in imaging tool within this VOI). Correlations between dopaminergic and glucose metabolism were calculated.

## Results

### Clinical Data

Overall, 32 pairs of matched AR patients (means: age 57 yrs, 24 male, Hoehn & Yahr stage 2.2, disease duration 4.5 yrs, LEDD 254 mg) and TD patients (means: age 56 yrs, 18 male, Hoehn & Yahr stage 1.8, disease duration 4.6 yrs, LEDD 231 mg) were identified. Patients of both subtypes were comparable regarding age, age at onset, Hoehn & Yahr stage, disease duration, LEDD and gender distribution (p>0.05 Wilcoxon-Mann-Whitney). Patients were matched as one aim of this study was to present different pathophysiologic aspects of PD subtypes. Therefore, it was from our point of view crucial to match patients which are per se comparable. This means to compare patients of the same disease duration, disease severity or LEDD. As far as are aware, there is no scaling factor or a non-linear approach to compare the progress of different PD-subtypes. Thus, we think that our approach of matching subtypes for multiple factors gives the best opportunity to relate these diverse disease entities. The clinical details are summarized in table 1.

**Table 1:** Patients’ characteristics.

	AR	TD
N	32	32
age	57±10.1	56±11.7
age at onset	53±11.4	51±12.3
Male/female	24/8	18/14
Hoehn & Yahr	2.2±0.9	1.8±0.9
Disease duration	4.5±4.3	4.6±4.9
LEDD	254±322	231±287

### SPM Analysis

As expected, the SPM analysis for the F-dopa-PET using an independent samples t-test revealed a significant difference (p = 0.016, set level) in the caudate and anterior putamen indicating a reduced dopaminergic uptake for AR compared to TD patients (see figure 1 top).

### Global and Regional Metabolism

The global FDG-metabolism was not significantly different between subgroups (AR: 25.26±4.77 µmol/100 g/min vs. TD: 27.41±3.99 µmol/100 g/min, p>0.05). The nCMRGlc in the striatal volume of interest (VOI) using the SPM mask for the dopaminergic subtype-difference showed a statistically significant difference (p = 0.013) between AR (1.1359 µmol/100 g/min) and TD patients (1.1802 µmol/100 g/min) (see figure 1 bottom), indicating lower glucose metabolism in the striatal areas of AR patients compared to TD patients. Likewise, the F-dopa SOR-1 values differed significantly between AR (SOR-1∶0.8597) and TD (SOR-1∶1.0006) patients (p = 0.025).

Additionally, contrasting the glucose and dopaminergic metabolism for both subtypes (see figure 2), the individual values of AR and TD patients revealed a positive correlation of the individual normalized FDG values (µmol/100 g/min) and dopamine uptake (SOR) (correlation *r* = 0.439, *p* = 0.005 for the overall group). This relationship was more pronounced for the AR patients, indicating a lower dopaminergic uptake is going along with a reduced glucose metabolism in this subtype of PD patients (AR: Pearson correlation *r* = 0.537, *p* = 0.032; TD: *r* = 0.110, *p* = 0.607; see figure 2).

## Discussion

Due to dual tracer imaging in identical PD patients, the present data allow an interpretation of the in-vivo substrates of the clinical phenomenology. We could prove a direct interaction of striatal dopaminergic and glucose metabolism in different subtypes of PD. To the best of our knowledge, this study demonstrates for the first time a concordantly reduced dopamine and glucose metabolism in the anterior striatum for AR patients. These results underline the importance of subgrouping PD patients. The underlying pathophysiological processes remain, however, elusive.

### Subtype-specific Pathophysiology

The main pathologic feature of PD is a dopaminergic (DA) neuron loss in the substantia nigra pars compacta. This results in DA denervation of the striatum, which in turn induces a demodulation of complex motor circuits comprising the motor and premotor cortices, the putamen, the globus pallidum, the subthalamic nucleus, and the thalamus [15]. Although this circuitry being understood as the main underlying basis for the development of bradykinesia and rigidity, its exact neural mechanisms remain incompletely recognized. Tremor in contrast, is supposed to be originated in the complex interplay of striato-cerebello-thalamic pathways [16].

### Structural and Functional Imaging of Subtypes

Structural changes with respect of white matter alterations in MRI scans offer conflicting results, suggesting no relevant overall impact of white matter hyperintensities (e.g. [6]). Yet, cerebellar or thalamic grey matter atrophy has been assigned to tremor development in PD [17] and distinct grey matter atrophy patterns have also been shown for PIGD patients [18], [19]. Kassubek et al. demonstrated an increased thalamic grey matter density as a correlate to a contralateral tremor in PD patients [20]. Nevertheless, as structural changes in both subtypes are not conclusive, other methods were developed during the past decades. Thereby, major insights into possible different aspects of the disease have been achieved through functional imaging. Our group – beside others – could repeatedly demonstrate PD subtypes showing differences in the dopaminergic uptake [7], [21], [22]. The underlying causes for these changes in the presynaptic dopamine metabolism might be a subtype-specific deafferentation as a consequence of different projections of nigral dopaminergic neurons to striatal structures as proposed by Jellinger et al. [23]. Hence, the present results of differences in the dopamine metabolism across both subtypes is in good accordance to the role of dopamine depletion in the development of akinesia and rigidity in AR patients.

Generally speaking, there is some evidence that PD patients show elevated levels of glucose metabolism in striatal regions compared to healthy controls as repeatedly demonstrated by different PET studies using partly no absolute quantification [9], [22], [24]. However, there is an ongoing debate about the significance of these data [25] and, additionally, these findings might be limited as studies using arterial input function in order to determine absolute quantification have not constantly shown a striatal upregulation [26], [27].

The increase in striatopallidal synaptic terminal activity was suspected as a result of nigrostriatal DA denervation [28]. However, one considerable weakness of the aforementioned studies was not taking into account relevant differences of subtype specifics, as none of the cited reports did a subgrouping into AR or TD patients. In a further study, Mure et al. described the PDTP as a distinct pattern of regional glucose metabolism in 9 tremor patients [10]. However, contrasting the PDTP with a general PD population (PDRP), an overlap was found for putaminal subregions [10]. The absolute differences in terms of nCMRGlc were not reported due to methodological differences. Additionally, the statistical techniques (principal component analysis) evolving these metabolic patterns are somewhat different to our chosen approach (SPM statistics, VOI-based analysis). Thus, major methodological differences may cause the different results in a multilevel dimension.

Considering metabolic substrates of bradykinesia and tremor, Lozza et al. reported in a FDG-PET study the severity of bradykinesia being correlated with higher CMRGlc in the putamen and globus pallidus whereas tremor scores related to lower CMRGlc in the putamen and cerebellar vermis. Both regions showed an extensive zone of overlap in the putamen bilaterally. This overlap was computed for each clinical variable independently and did not reflect colinearity.

### The Ventral Striatum and its Association to AR Patients

The prominent circumscribed reduced metabolism in AR patients was found in the ventral striatum, namely the caudate and the anterior putamen. The anterior putamen was already defined as a subtype-discriminating region in an earlier FP-CIT study of our own group [8]. Assumably, this reflects the region with the largest longitudinal subtype-specific change-index as the posterior putamen is prone to be affected early in the course of the disease [29], [30]. The significant correlation of the FDG and F-dopa uptake in this region in AR patients is another evidence for a subtype-specific feature. The caudate has been linked repeatedly to cognitive dysfunction [31], [32]. AR patients tend to have a more pronounced cognitive decline compared to TD patients [33]. The combination of dopaminergic imaging (dopamine transporter) and FDG-PET was recently applied in a study of Niethammer et al. [34] to focus on the link between caudate dopaminergic functioning and cognition in PD. This revealed a direct correlation between DAT binding and Parkinson’s disease cognition-related pattern (PDCP) expression values. The interaction between motor function and striatal dopamine−/glucose-metabolism was not considered in data analysis. Taken together, the pronounced reduced metabolism in the ventral striatum might be reflected, among other things, through the impaired cognitive functioning as one symptom complex of AR patients. Unfortunately, neuropsychological parameters which may have explained some of our own results between subgroups, were not available and should be obliged to further research.

### Strength and Limitations

The main strength of this manuscript is the dual tracer approach in a large group of PD patients using quantified & dynamic sets of PET-scans allowing a distinct differentiation of individual glucose/dopaminergic uptakes. The large number of individually characterized patients gives the unique opportunity to detect subtype-specific differences on different pathophysiological levels (glucose and presynaptic dopaminergic metabolism).

Previous dual tracer studies employed the combination of dopaminergic and metabolic imaging [35], [36]. They illustrated a relationship between striatal dopaminergic decline and concurrent whole brain metabolic changes in parkinsonian patients (e.g. in thalamus, cerebellum or parieto-occiptal regions). However, neither approach focussed on a direct association of striatal dopaminergic or glucose metabolism or considered subtypes of PD. The present approach does not consider the network components in the emergence of bradykinesia or tremor. Especially the cerebello-thalamo-cortical pathways are not further accounted for in this analysis. Thus, these data allow only an implicit gain of knowledge on one part of the motor network. However, the main goal of this study was to assess the direct, local influence of dopaminergic input on the striatal nuclei. As we presently cannot make any statement about the direct relationship between local striatal dopaminergic uptake and remote cortical regions (e.g. in terms of connectivity), we decided to focus on the local glucose metabolism rather than a whole brain analysis. Future multimodal imaging techniques (e. g. by tractography analysis or diffusion tensor imaging) may overcome this obstacle in terms of combining local and wholebrain metabolism.

Beside the weaknesses of a retrospective analysis, the missing UPDRS part III is a main flaw of this study. However, the rigorous exclusion of patients in any doubt of correct assignment to one subtype reduces the risk of an incorrect classification. More than 70% of the patients were excluded due to this procedure. We are rather confident that both the strong experience in this movement disorders center and the strict inclusion criteria based on the charts – as described above - supports an assignment of patients to the correct subtype. However, the lack of UPDRS subscores comprises the risk of an inaccurate classification of patients. As the results for the dopaminergic metabolism are in line with previous findings [7], [8], this constraint might be relative.

We had no structural data (e.g. MRI scans) available in these patients. Thus, we could not compare the regional hyper- or hypometabolism with structural gray matter atrophy or white matter hyperintensities as shown before [6], [19]. The linkage between MRI- and PET−/SPECT-imaging is apparently essential to elucidate the underlying pathophysiological processes between structure and function in distinct PD subtypes.

## Conclusion

The present findings indicate a different involvement of both presynaptic dopaminergic uptake and glucose metabolism in the ventral striatum for AR and TD subtypes of PD. These results suggest a differential involvement within distinct regions of the basalganglia-cerebello-thalamocortical circuit in these subgroups. Future research is needed to clarify these observations and achieve the longitudinal progress of subgroups.

## Supporting Information

File S1 **Methodological considerations.**
(DOCX)
