# Supplementary material for: Parkinson’s Disease Subtypes Show a Specific Link between Dopaminergic and Glucose Metabolism in the Striatum
Source: PLoS One. 2014 May 21;9(5):e96629. doi: 10.1371/journal.pone.0096629 (PMC4029550; doi:10.1371/journal.pone.0096629)
Supplement: File S1 — Methodological considerations. (DOCX) [file pone.0096629.s001.docx]

**S1: Methodological considerations**

Figure 2 shows the regional uptake of FDG and F-dopa in different subtypes of PD. The total number of included patients was lower than in the evaluation of the SPM analysis and the resulting VOI analysis of the FDG scans as presented in figure 1.

This methodological shortcoming is due to different analytic steps: for the SPM analysis we used summed F-dopa scans from the 9 frames. These data had a sufficient striatal and extrastriatal uptake for an exact normalization procedure in SPM onto a FP-CIT-template.

The F-dopa SOR images were generated from the last frame (80-90 min after injection). The resulting striatal and extrastriatal uptake in these scans was rather low, so that in 24 from 64 patients the normalization procedure failed. For this reason, we included only the remaining 40 patients for a further evaluation and contrast of glucose and dopamine uptake as presented in figure 2.
